# Multiple Target-Specific Molecular Agents for Detection and Image Analysis of Breast Cancer Characteristics in Mice

**DOI:** 10.2174/1566524011313030014

**Published:** 2013-03

**Authors:** S Ke, W Wang, X Qiu, F Zhang, J.T Yustein, A.G Cameron, S Zhang, D Yu, C Zou, X Gao, J Lin, S Yallampalli, M Li

**Affiliations:** 1 Department of Radiology, Baylor College of Medicine, One Baylor Plaza, Houston, Texas 77030, USA; 2 Orthopedic Surgery Center, Tang-Du Hospital, The Fourth Military Medical University, Xi’an, Shaanxi, 710038, P.R. China; 3 State Key Laboratory of Oncology in South China, Department of Imaging and Interventional Radiology, Cancer Center, Sun Yat-sen University, Guangzhou, Guangdong, 510060, P.R. China; 4 Department of Pediatric Hematology & Oncology, Texas Children’s Hospital, Baylor College of Medicine, One Baylor Plaza, Houston, Texas 77030, USA; 5 Department of Molecular and Cellular Oncology, The University of Texas M.D. Anderson Cancer Center, 1515 Holcombe Blvd, Houston, Texas 77030, USA; 6 Department of Biochemistry and Molecular Biology, Harbin Medical University, Harbin, Heilongjiang, 150081, P.R. China; 7 Department of Arteriosclerosis, Capital Medical University, Beijing Anzhen Hospital, Beijing, 100029, P.R. China; 8 The Vivian L. Smith Department of Neurosurgery, Department of Integrative Biology & Pharmacology, The University of Texas Health Science Center at Houston, Houston, TX 77030, USA

**Keywords:** CT, EGF, FDG, Her-2, interleukin-11, MMP, molecular imaging, multi-agent imaging, multi-modality imaging, optical imaging, PET, RGD, SPECT.

## Abstract

Breast cancer is a heterogenetic tumor at the cellular level with multiple factors and components. The inconsistent expression of molecular markers during disease progression reduces the accuracy of diagnosis and efficacy of target-specific therapy. Single target-specific imaging agents can only provide limited tumor information at one time point. In contrast, multiple target-specific imaging agents can increase the accuracy of diagnosis. The aim of this study was to demonstrate the ability of multi-agent imaging to discriminate such differences in single tumor. Mice bearing human cancer cell xenografts were tested to determine individual differences under optimal experimental conditions. Neovasculature agent (RGD peptide), tumor stromal agent (matrix metalloproteinase), and tumor cell markers (epidermal growth factor, Her-2, interleukin 11) imaging agents were labeled with reporters. ^18^F-Fluorodeoxyglucose was used to evaluate the tumor glucose status. Optical, X-ray, positron emission tomography, and computer tomography imaging modalities were used to determine tumor characteristics. Tumor size and imaging data demonstrated that individual differences exist under optimal experimental conditions. The target-specific agents used in the study bind to human breast cancer cell lines *in vitro* and xenografts *in vivo*. The pattern of binding corresponds to that of tumor markers. Multi-agent imaging had complementary effects in tumor detection. Multiple noninvasive imaging agents and modalities are complementary in the interrogation of unique biological information from each individual tumor. Such multi-agent approaches provide methods to study several disease components simultaneously. In addition, the imaging results provide information on disease status at the molecular level.

## INTRODUCTION

Breast cancer development involves interactions of tumor cells, tumor stroma, factors in the genetic background of the patient, and environmental influences [1-5]. Locally, breast cancer cells have the ability to adapt their microenvironment and survive under varying conditions, even during chemotherapy. This suggests that multiple tumor components are constantly changing to maintain proliferation and metastasis of the cancer cells. The genomic instability and DNA mutation of breast cancer cells during multistep tumorigenesis process affect the expression of tumor markers and manifest in various histological and clinical types [6-8]. Accuracy of diagnosis may be markedly reduced by cellular heterogeneity of breast cancers and unpredictable behavior of molecular markers. A combination of serum markers, genetic fingerprint, and target-specific molecular imaging may provide improvements in diagnosis and evaluation of responses to treatment [9]. Recent clinical trials have demonstrated the benefits of multiple target-specific imaging approaches to evaluate the risk of treatment resistance and poor outcome. The results not only show that such an approach is feasible but more importantly, that unique biological information on individual tumors can be detected by the method [10, 11]. The clinical studies confirmed that tumor progression is a dynamic process, and the same tumor shows biological variability at any one time point [10].

Use of multiple target-specific agents to determine disease markers and eventually make a correct diagnosis has been a standard practice in clinical medicine, especially in pathological immunohistochemistry (IHC), since 1964 [12]. Everyday, physicians order many laboratory tests to determine status of disease surrogate markers to improve diagnosis and treatment for each patient. The usefulness of surrogate markers led the United States Food and Drug Administration (FDA) in 2009 to approve the first protocol that made use of five surrogate molecular markers to identify and diagnose potential malignancies for surgical treatment of ovarian cancer [13]. Other clinical studies have also suggested that surrogate markers are useful to determine proliferation index in breast cancer [14]. The application of a multiple surrogate markers approach in IHC requires that tumor tissue be divided into multiple samples, each of which can then be tested in parallel with one or multiple antibodies having different optical reporters. However, obtaining tumor tissue samples requires invasive procedures, which may increase the risk to the patient. On the other hand, current improvements in molecular imaging technology are providing alternative noninvasive approaches to accurately study disease status in a longitudinal manner. Molecular imaging uses the same target-specific components as IHC. Imaging technology permits the analysis of disease status at the level of the entire body, the lesion, and the cell, and minimizes sampling error while permitting simultaneous analysis of multiple disease factors.

Nuclear medicine remains the gold standard of molecular imaging for both the clinic and research laboratory. Optical molecular imaging is a rapidly advancing imaging modality that allows simultaneous detection of multiple disease targets in preclinical area. Both nuclear and optical imaging modalities are the most sensitive in terms of detection of molecular events.

In this study, we generated replicate xenograft tumors from cell lines in nude mice under optimal experimental conditions to test for individual tumor/host differences among the mice. We prepared and validated multiple target-specific agents to detect different breast cancer molecular components. Specifically, we investigate the ability of labeled RGD peptide agent to bind areas of neovasculature, matrix metalloproteinase (MMP) peptide to tumor stroma, binding of epidermal growth factor (EGF), Herceptin, and interleukin 11 (IL-11) peptide agents to their corresponding receptor-positive tumor cells. We then analyze the collected image data in various combinations to explore complementarity in methods.

## MATERIALS AND METHODS

### Cell Lines

Human cancer cell lines were purchased from the American Type Culture Collection (Manassas, VA), transfected with the luciferase gene, and then grown in culture in Dulbecco’s Modified Eagle’s Medium with high glucose or F12 medium (Invitrogen, Carlsbad, CA), supplemented with 10% fetal bovine serum (Hyclone, Logan, UT), in incubators with 5% CO_2 _at 37°C.

### Tumor Xenografts

Female nude mice (4- to 6-week-old, 18-22 g, n=65) (Harlan, Indianapolis, IN) were maintained in a pathogen-free mouse colony in a facility accredited by the American Association for Laboratory Animal Care (Accredited Facility Number: 876), and all experiments were performed in compliance with the guidelines of the Institutional Animal Care and Use Committee (Approved Protocol Number: AN-4239). For tumor implantation, cultured tumor cells were harvested near confluence by treating monolayers with 0.05% trypsin-ethylenedinitrilotetraacetic acid. Cells were pelleted at 130 × *g* for 5 min and resuspended in sterile phosphate-buffered saline. Approximately 1 × 10^6^ cells were implanted subcutaneously into each mouse.

### Imaging Agent Synthesis

All agents were designed and synthesized in-house as previously described [15-20]. They were purified by high-performance liquid chromatography (HPLC) and confirmed by mass spectrometry, analytic HPLC, and fluorescent spectrophotometry. The optical/nuclear dual-labeled IL-11 agent was tagged with a second label, ^111^InCl_3_ (PerkinElmer Life and Analytical Sciences, Billerica, MA) or ^64^Cu (Washington University Medical School, St. Louis, MO). ^18^F-FDG was purchased from Cyclotope (Houston, TX).

### Binding and Blocking Reactions

The human breast cancer cell lines MDA-MB-231, MDA-MB-468, and SKBr3 were used for *in vitro* binding analysis. All blocking assays were performed by pre-incubating cells with a 200-fold excess of blocking antibodies in 0.2 ml of culture medium for 45 min at 37°C, followed by addition of the imaging agents. Cells were fixed and counterstained with 1 µM Sytox Green (Invitrogen) in 95% ethanol for 15 min at 4°C. IL-11 protein Neumega was purchased from Wyeth Pharmaceuticals, Inc. (Oprelvekin, Philadelphia, PA). MMP antibodies were purchased from Millipore (Billerica, MA).

### Confocal Microscopic Imaging

Stained cells were transferred to slides for microscopic examination. Images were captured with an Olympus confocal microscope (Fluorview 1000, Olympus America, Center Valley, PA). Near-infrared (NIR) dyes were measured at excitation/emission (Ex/Em) wavelengths of 765/810 nm and cell nuclei at 488/510 nm. Signal intensities were recorded from one slice of multiple z-stacks with 0.5-µm gaps. Sytox green and imaging agents or NIR dye signals were pseudocolored green (Em 510 nm) or red (Em 810 nm), respectively.

### Animal Imaging

Tumors developed after 3 to 4 weeks of growth in the implanted mice to 8-15 mm in diameter. Tumors were visualized by intraperitoneal injection of 3 mg VivoGlo Luciferin (Promega, Madison, WI), and pseudocolored cyan. Imaging agents (2-10 nanomol) were injected into the tail vein of anesthetized mice. Mice were imaged immediately after injection and for as long as 48 hours afterward. Optical and X-ray images were recorded by Kodak In-Vivo Multispectral System FX (Carestream Health Molecular Imaging, New Haven, CT). *In vivo* positron emission tomography (PET)/single photon emission computed tomography (SPECT)/computed tomography (CT) imaging was performed on a Siemens MicroCAT II SPECT/CT and Inveon PET (Siemens Medical Solutions, Malvern, PA).

### Statistical Analysis

SAS software v9.1 (SAS Institute, Cary, NC) was used to analyze data by one-way ANOVA or the general linear model. Data comparison was presented in notched box-and-whisker plots. The medians (central lines) of two box-and-whisker plots were considered to be significantly different at the 0.05 level (95% confidence) if the corresponding notches did not overlap.

## RESULTS

### Identification of Tumor/Host Differences

**Fig. (1):**
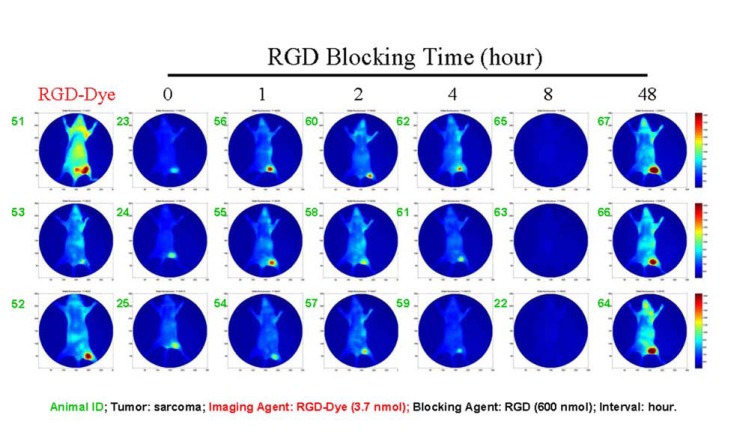
Illustration of individual differences under optimal experimental conditions. Despite all mice being housed and treated under the same conditions, and injected with identical doses of blocking agent and target-specific imaging agent, the sizes of tumors and intensity of imaging varied between the animals.

Tumor growth differences and animal responses to blocking agent are presented in Fig. (**1**). No two tumors in all 21 mice had the same size. Within the blocking group, each of the three mice that were tested responded differently except for the single group that was blocked for 8 hours. Even for the group simply injected with imaging agent alone (Fig. **1**, RGD-Dye), the three mice responded differently. The imaging agent strongly bound to the tumor of mouse 51. However, this mouse also exhibited the highest background signal intensity in this group, as shown by high signal intensity over the whole body and bladder. Similar phenomena could be found in all other blocking groups (Fig. **1**, panels 0-4 and 48).

The data demonstrate individual differences presented even under optimal experimental conditions since all mice were sequentially injected with an identical dose of blocking agent and an identical dose of target-specific imaging agent. All mice were treated at the same time and maintained under the same conditions. Tumor cell inoculations of all mice were performed on the same day using the same suspension of cells. All data were analyzed under the same conditions.

### *In Vitro* Imaging

**Fig. (2):**
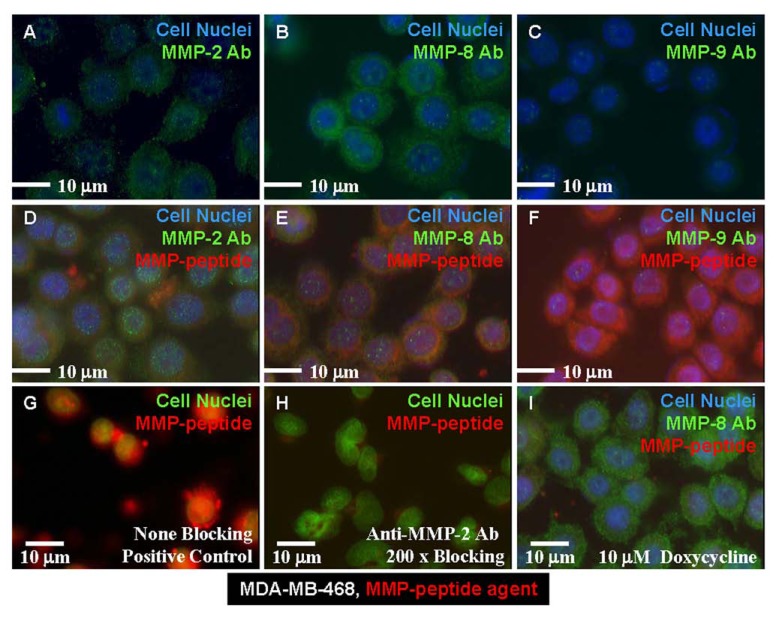
MMP cell images. Human breast cancer cell line (MDA-MB-468) was positive for MMP-2 expression (**A**), strongly positive for MMP-8 (**B**), and weakly positive for expression of MMP-9 (**C**). (**D**) The MMP-peptide agent had the same binding site as did the anti-MMP-2 Ab. (**E**) The same peptide had a different binding site than that of the anti-MMP-8 Ab. (**F**) MMP-peptide bound to different motif than MMP-9 Ab. (**G**) The peptide agent bound to none of the positive control cells that had been treated with blocking Ab. (**H**) Anti-MMP-2 Ab blocked the peptide binding to the cells. (**I**) Doxycycline-treated cells lost the capability of binding to the MMP peptide agent, but were still able to bind the anti-MMP-8 Ab.

Binding of the MMP agent to the breast cancer cell line MDA-MB-468 is shown in Fig. (**2**). This cell line is positive for MMP-2 (Fig. **2A**), strongly positive for MMP-8 (Fig. **2B**), and weakly positive for MMP-9 (Fig. **2C**). After co-incubation with anti-MMP antibodies (Ab), our MMP-peptide agent bound weakly in the presence of MMP-2 Ab condition at the same location (Fig. **2D**, yellow), showed positive binding in the presence of MMP-8 Ab at a different location (Fig. **2E**, yellow), and strong binding in with MMP-9 Ab (Fig. **2F**). In a side-by-side comparison study, the MMP-peptide agent in MDA-MB-468 cells presented a strong binding signal intensity (Fig. **2G**). In contrast, pre-incubation with 200-times excess of anti-MMP-2 Ab almost completely blocked the peptide agent binding (Fig. **2H**). Pretreatment of the cells with a nonspecific MMP inhibitor, doxycycline, did not inhibit MMP-8 Ab binding to the cells, but these cells lost the ability to bind the MMP-peptide agent (Fig. **2I**). These data suggest this peptide agent has a related binding locus to that of MMP-2 Ab, a different binding mechanism than MMP-8 Ab, and different binding location than MMP-9 Ab.

**Fig. (3):**
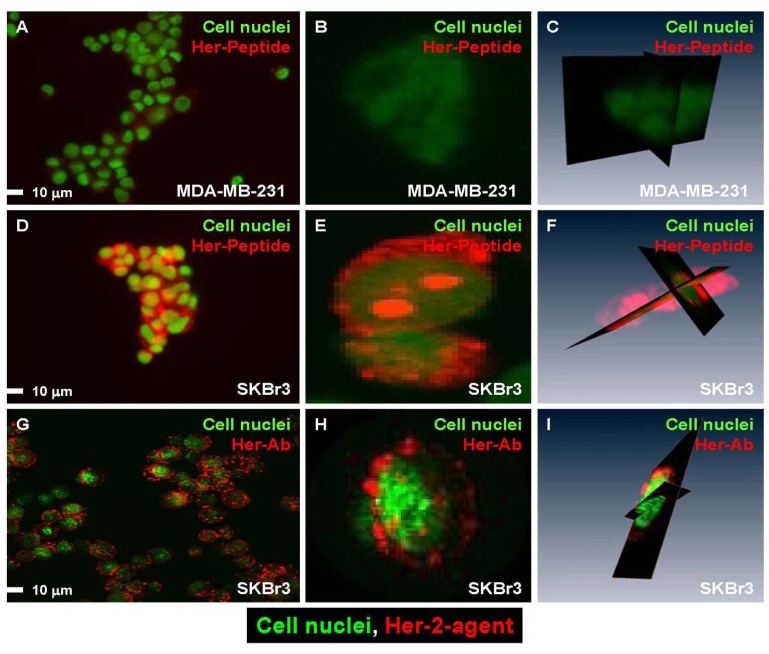
Side-by-side confocal images comparing the Her-2 specific imaging agents. (**A**) The Her-2 peptide agent did not bind to MDA-MB-231 cells, which had the lowest expression of Her-2 relative to the other two cell lines. (**B**, **C**) Single-cell 2-D and 3-D image confirming that the agent was not present in the cells. (**D**) The Her-2 peptide agent bound to receptor-positive cells. (**E**, **F**) Single cell images demonstrated the agent not bound to the cell membrane and in the cytosol but also in cell nuclei. (**G**) Reporter labeled Herceptin bound to receptor-positive cells. (**H**, **I**) Single cell images show that the Ab agent only bound to the cell membrane but was not internalized. The images also demonstrated the imaging agents binding sites were not evenly distributed within the cell.

Her-2 agents were tested on the receptor-negative cell line MDA-MB-231 (Fig. **3A-C**) and receptor-positive cell line SKBr3 (Fig. **3D-I**). Fig. (**3**) presents the cell binding results of peptide (Fig. **3A-F**) and Ab (Fig. **3G-I**) agent. A side-by-side study was performed to compare the cellular distribution of both Ab and peptide agents on receptor-positive and -negative cells. The Her-2 peptide agent did not bind to most of the receptor-negative cells (Fig. **3A**), and the result was supported by single-cell confocal images (Fig. **3B**, **C**). This peptide agent bound to SKBr3 cells (which are positive for all receptors tested) (Fig. **3D**) and was internalized into the cell (Fig. **3E**, **F**, confocal images). In contrast, the Her-2 Ab agent bound to most receptor-positive cells (Fig. **3G**) but was not internalized (Fig. **3H**, **I**, confocal images).

**Fig. (4):**
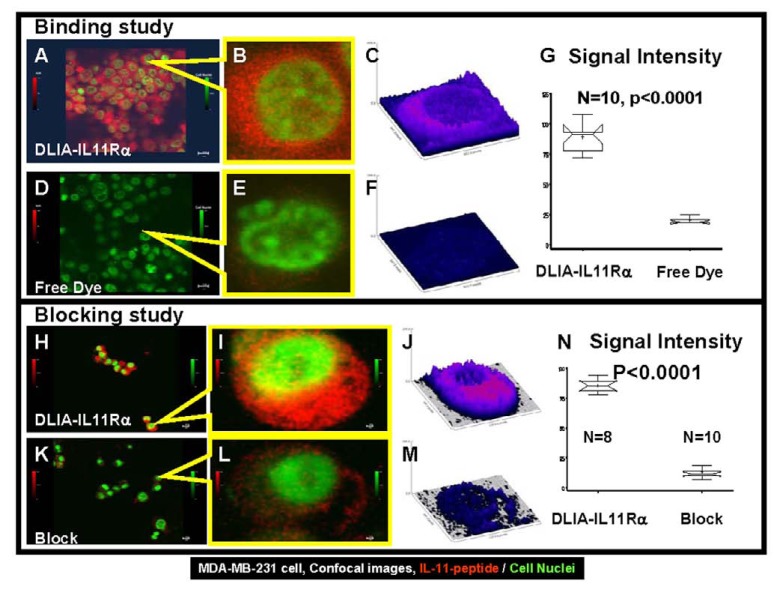
Side-by-side confocal images showing distribution of DLIA-IL11Rα, free NIR dye, and blocked signals in cancer cells. (**A**, **D**): Merged NIR (DLIA-IL11Rα or free dye) and cell nuclei images in the population view. (**B**, **E**): Merged single-cell images of cell nuclei (DLIA-IL11Rα) and NIR. (**C**, **F**): Comparison of NIR signal intensity between DLIA-IL11Rα or free dye from single-cell images. (**G**) Statistical comparison of DLIA-IL11Rα and free dye. (**H**, **K**): Merged NIR (DLIA-IL11Rα- or Neumega-blocked) and cell nuclei images in the population view. (**I** and **L**): Merged single-cell images of nuclei and NIR (DLIA-IL11Rα- or Neumega-blocked). (**J**, **M**): Comparison of NIR signal intensity in blocked (Neumega) *vs* unblocked DLIA-IL11Rα reactivity in single cells. (**N**): Statistical comparison of signal intensity in blocked (Neumega) *vs* unblocked DLIA-IL11Rα reactivity in samples. The cells in the images are MDA-MB-231.

The binding of the dual-labeled optical/nuclear IL-11 imaging agent (DLIA-IL11Rα) was tested on IL-11 receptor-positive MDA-MB-231 cells and measured by confocal microcopy. Fig. (**4**) shows a side-by-side confocal image analysis comparing the binding of free NIR dye, blocking effects, and DLIA-IL11Rα signals at both the population and single-cell levels. The binding of DLIA-IL11Rα to IL-11 receptor-positive cells is shown in Fig. (**4A-C**). The cell binding of the free NIR dye is shown in panels D-F. The images show cell nuclei in green and DLIA-IL11Rα or free NIR dye bound to cells in red. Fig. (**4A**, **D**) shows the merged cell nuclear and NIR images for free NIR dye and DLIA-IL11Rα in the population view. Fig. (**4B**, **E**) shows merged single cell views, while Fig. (**4C**, **F**) shows the NIR signal intensity. These data demonstrate that the NIR signals are located within the cell membranes, and the signal from DLIA-IL11Rα is much stronger than that from free NIR dye (*P *< 0.0001, Fig. **4G**).

The blocking effects of population and single-cell images are shown in Fig. (**4H-N**). The merged images show much stronger DLIA-IL11Rα binding to cells in the unblocked control (panels H and I) than in the cells pre-incubated with IL-11 protein Neumega in Fig. (**4**), panels K and L. The single-cell NIR signal intensity plots (Fig. **4J**, **M**) show the differences in the unblocked control of DLIA-IL11Rα and the blocking effects of Neumega in the same imaging setting. This difference, due to blocking of the target, is statistically significant (*P* < 0.0001, Fig. **4N**).

### *In Vivo* Imaging

**Fig. (5):**
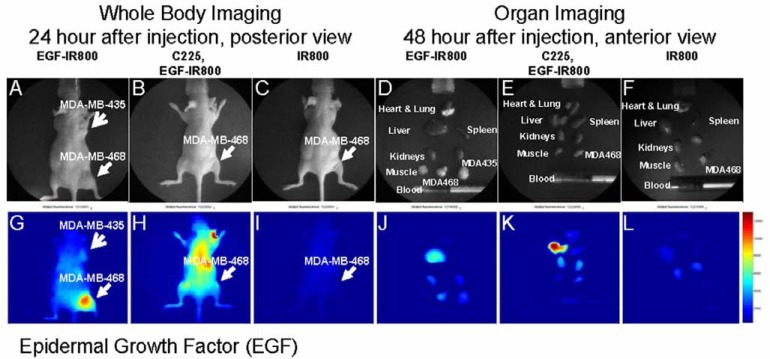
*In vivo* imaging of EGF reactivity in human breast cancer xenografts. Photographs of the whole bodies of the mice (**A-C**) and dissected organs (**D-F**) taken with visible light are shown in the top row. The bottom row shows the corresponding NIR images (**G-L**). (**A**) Visible light image showing the location of EGFr-negative (MDA-MB-435) and -positive (MDA-MB-468) tumors. (**B**) Receptor-positive tumor blocked with C225 Ab. (**C**) Receptor-positive tumor injected with dye. (**D**) Visible light image of the mouse, and dissected organs, bearing both receptor-positive and -negative tumors. (**E**) The organ image of the mouse blocked with Ab then injected with target-specific imaging agent. (**F**) Dissected organs of the mouse injected with NIR dye alone. (**G**) EGF imaging agent showed stronger signal intensity in the receptor-positive tumor (MDA-MB-468) than the receptornegative tumor (MDA-MB-435). (**H**) Blocking with the C225 Ab in receptor-positive tumor cells reduces the signal intensity. (**I**) There is no detectable signal in the receptor-positive tumor injected with dye alone. (**J**) Organ imaging shows higher signal intensity in the receptor-positive tumor than the receptor-negative tumor. The liver also shows a high signal intensity, as seen in the imaging agent distribution pattern. (**K**) The C225 Ab blocked binding of the EGF agent to the receptor-positive tumor. (**L**) NIR dye did not bind to the receptor-positive tumor.

Fig. (**5**) shows the imaging results of the EGF agent on both EGF receptor (EGFr)-positive MDA-MB-468 and the receptor-negative MDA-MB-435 tumors. The visible light images show the tumor location in the whole animal (Fig. **5A-C**, arrows) and the dissected organs (Fig. **5D-F**). Mice injected with EGF imaging agent showed higher signal intensity in the MDA-MB-468 tumor than the receptor-negative MDA-MB-435 tumor (Fig. **5G**). The imaging signal intensity decreased when the receptor-positive tumor was treated with specific Ab C225 (Fig. **5H**). Mice bearing receptor-positive tumors and injected with dye alone showed very low signal intensity in whole body imaging, and there was no increased signal in the tumor region (Fig. **5I**). The organ image showed the signal intensity was high in the liver and receptor-positive tumor region. Both kidneys and receptor-negative tumors showed weak signals from imaging reporter (Fig. **5J**). The Ab-blocked receptor-positive tumor showed a significant decrease in signal intensity (Fig. **5K**) relative to the unblocked positive control, while no detectable signal was seen in the receptor-positive tumor injected with dye alone (Fig. **5L**).

Quantitative analysis of the tumor-to-background ratio (TBR) is plotted in Fig. (**6**). The receptor-positive tumor cell line MDA-MB-468 injected with EGF-IR800 imaging agent had a significantly higher TBR than Ab-blocked receptor-positive tumors (*P* < 0.05), injection with dye alone (*P* < 0.05), and the receptor-negative tumor (MDA-MB-435; *P *< 0.05).

**Fig. (7):**
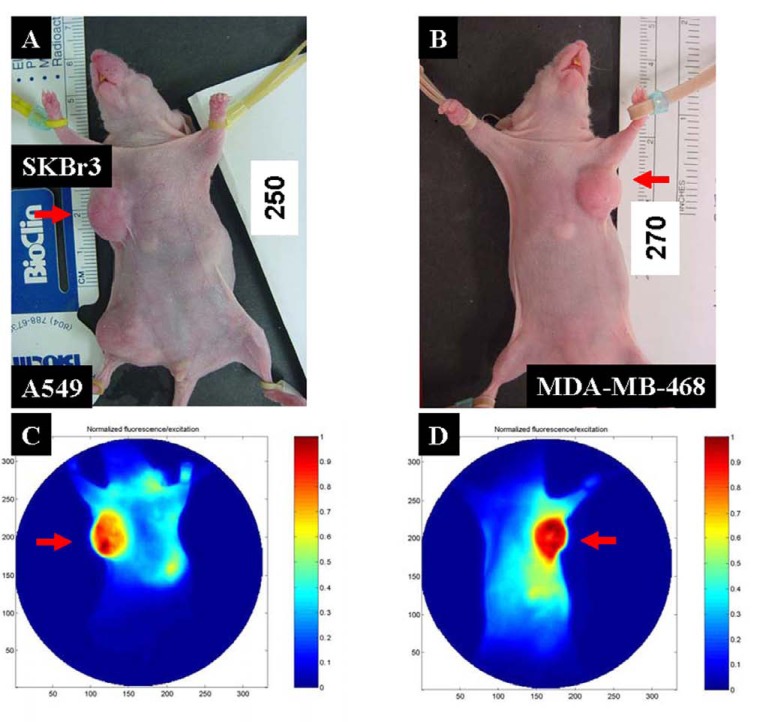
MMP agent imaging of human breast cancer xenografts. (**A**) Visible images showing the location of the tumors and the MMP-positive tumors (SKBr3), indicated by red arrows. (**B**) Visible image showing the location of location of human breast cancer xenograft of MDA-MB-468 cells. (**C**) NIR image showing that the MMP-positive tumor had a higher signal intensity than MMP-negative tumor (A549). (**D**) The MMP agent shows strong binding to the MDA-MB-468 cell xenograft tumor. (For interpretation of the references to color in this figure legend, the reader is referred to the web version of this paper).

MMP agent imaging of the breast cancer xenograft results is shown in Fig. (**7**). The location of breast tumors are shown in the color images (Fig. **7A**, **B**), indicated by red arrows. Both SKBr3 (Fig. **7C**) and MDA-MB-468 (Fig. **7D**) tumors displayed a very high MMP signal intensity. Furthermore, the signal intensity was not evenly distributed in the tumor region. This uneven signal distribution inside tumors is reminiscent of the heterogeneity of molecular markers during tumor progression.

**Fig. (8):**
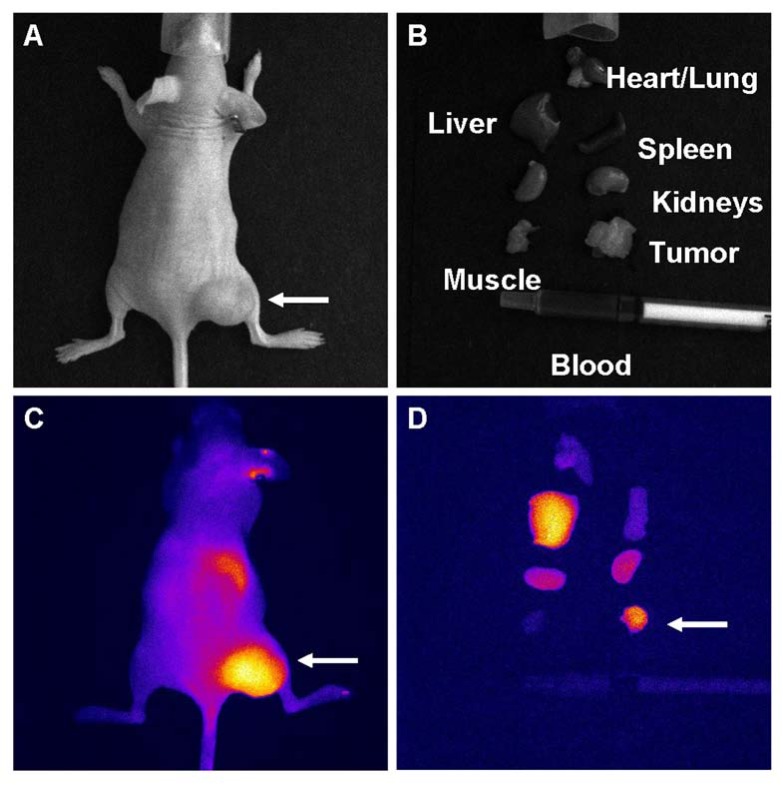
NIR dye-labeled Herceptin imaging results. (**A**) Visible light image showing the Her-2 positive tumor (SKBr3 cells). (**B**) Dissected organ display from the mouse in (**A**). (**C**) Whole body NIR image showing high signal intensity in the tumor, liver, and kidney region. (**D**) Dissected organ NIR image confirming the whole body imaging results.

NIR dye-labeled Herceptin imaging results are showed in Fig. (**8**). The SKBr3 tumor location is indicated by an arrow (Fig. **8A**), and dissected organs are pictured in Fig. (**8B**). Labeled Herceptin binding to the tumor is clearly visible in the whole body (Fig. **8C**) and organ (Fig. **8D**) images. Both images also show uptake of this antibody agent by the liver.

**Fig. (9):**
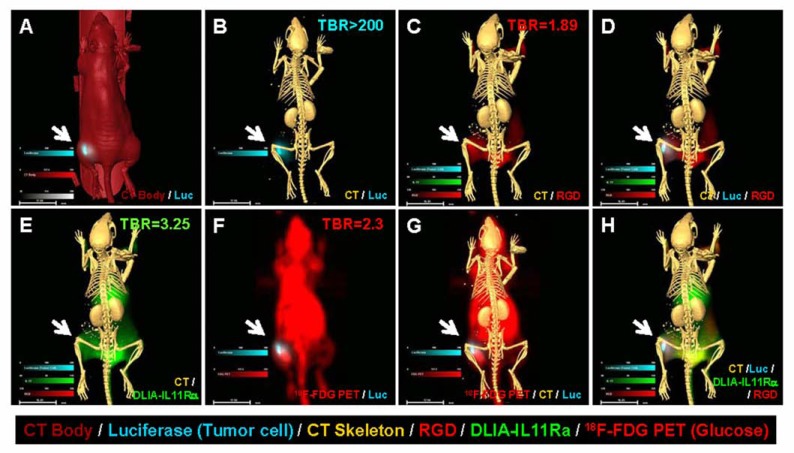
*In vivo* multi-agent images of human breast cancer xenografts. (**A**) Luciferase (blue) and CT body image (red) of MDAMB- 231 xenograft. (**B**) Luciferase (blue) cell growth pattern and CT skeleton image. (**C**) CT skeleton (yellow) and vasculature agent RGD (red) show the hypervascularization stage at the tumor site. (**D**) Merged luciferase, RGD, and skeleton image showing an uneven distribution of luciferase signal in the tumor. There is a positive correlation between luciferase and RGD agent signal intensities, suggesting that tumor growth requires neovascularization. (**E**) The human breast cancer xenograft has high DLIA-IL11Rα signal intensity. (**F**) ^18^F-FDG glucose uptake in luciferase-positive MDA-MB-231 xenograft. (**G**) Merged images of CT skeleton, 18F-FDG, and luciferase showing the tumor location and glucose uptake state. Note that some tumor cells remain luciferase-positive, but most have become luciferase-negative at this stage. (**H**) Merged image of CT skeleton, RGD, luciferase, and DLIA-IL11Rα staining demonstrates the relationship between of tumor location, tumor cell heterogeneity, neovasculature, and location of disease markers.

To demonstrate the role of IL-11 in breast cancer and the usefulness of the multi-agent imaging approach to detect multiple disease components, a group of mice were inoculated with luciferase-positive MDA-MB-231 cells. Fused images clearly demonstrate the relationship of each disease component (Fig. **9**). The CT body and luciferase image indicates an uneven distribution of the luciferase signal in the tumor mass (Fig. **9A**). The CT image of the skeleton and the luciferase image show that the tumor cells did not invade the bone (Fig. **9B**). The CT and RGD image demonstrates the anatomic location of the disease and the increased density of the vasculature (Fig. **9C**). The CT, luciferase, and RGD images show the heterogeneous tumor growth with formation of neovasculature around the tumor mass (Fig. **9D**). The CT and DLIA-IL11Rα images show that this tumor mass had a higher DLIA-IL11Rα signal intensity (Fig. **9E**). The ^18^F-FDG and luciferase image shows that the majority of the tumor mass had a higher glucose uptake, but that the cells were luciferase-negative (Fig. **9F**). The fused CT skeleton, ^18^F-FDG/PET, and luciferase image shows disease location, glucose status, and properties of the tumor cells (Fig. **9G**). The final merged CT, RGD, DLIA-IL11Rα, and luciferase image demonstrates the relationship among the four disease components (Fig. **9H**).

## DISCUSSION

Identifying individual differences is one of the most important challenges in the future for personalized molecular medicine. Our data show that even under highly controlled experimental conditions, tumor-bearing animals still exhibit unequal tumor sizes and specific signal intensities. Given that animal tumor grafts from a single cell line have much less variability than seen in human tumors, such variations are most likely due to the host (animal) response. In our study, individual differences existed within practically every treatment group and even in the control group. One mouse in the study appeared as if it might have a relatively weaker ability to eliminate unbound imaging reagent than the other mice, and another one a relatively stronger ability.

Individual differences can greatly influence tumor development, treatment outcome, and optimal imaging time. Considering how much variability we observed here under controlled conditions, one begins to understand why even greater variability in the clinical situation must exist, where we treat thousands of patients with diverse backgrounds with the same protocol. However, noninvasive imaging methods have the potential to identify such variation and applying treatments in ways that fit individual patient needs. Molecular imaging tools can be used for this purpose, allowing us not only to determine optimal doses and schedules of administration [21], but also detect variable disease manifestations.

Use of multiple disease markers to identify disease status at the molecular level is another factor critical for the future of personalized molecular medicine [22]. Human cancer is never homogenous in terms of cell type. Recent advances in breast cancer research have suggested that this disease has at least five subtypes [23] and the involvement of multiple gene mutations [24, 25] and molecular pathways [26, 27]. These developments provide more opportunities to understand the mechanisms underlying disease progression. It also demonstrates the complicity of breast cancer, which is therefore well suited to investigation by multiple imaging agents and modalities simultaneously.

Multi-agent molecular imaging approaches are not only feasible but complementary to each other. Such a noninvasive approach can detect each tumor's characteristics in more detail and accuracy over a significant time scale [10, 11]. As we have demonstrated in this study, MDA-MB-231 is a Her-2-negative cell line, and all agents specific target to Her-2 will yield negative results. The combination of IL-11 and RGD agents clearly demonstrated the possibility to detect the tumor mass containing Her-2-negative cells. The combination of RGD, MMP, and IL-11 agents may have a unique role in detecting molecular marker-negative tumors, such as triple negative breast cancer cells, since those agents are not targets on the tumor cell itself. Because multi-agent imaging targets on several factors on the tumor, both heterogeneity and unique properties of each tumor mass may be determined. Our previous data indicated that each tumor contains multiple disease components, and each component will change within a short time period [28]. This dynamic change has great impact on imaging and therapeutic results. As an example, a nonfunctional blood vessel could prevent both imaging and therapeutic agents from being delivered to the tumor region. Therefore, the imaging or therapeutic agent should be administered accordingly to achieve the best results.

Nuclear medicine is the most validated area in molecular imaging. Both PET and SPECT can detect low doses of injected tracers and provide 3D data [29]. Optical imaging is a rapidly growing preclinical molecular imaging modality that uses the same targets as nuclear molecular imaging, but replaces radioactive reporters with optical reporters. Optical imaging takes advantage of the broad light spectrum and narrow-band optical filters to separate multiple signals from different target-specific agents, allowing the simultaneous detection of multiple disease components without radiation exposure. Imaging studies can be performed at both the cellular and superficial whole-body levels [22].

Our confocal cell images demonstrate the uneven distribution of binding sites at the cellular level. The imaging results further suggest three challenges for quantitative molecular imaging. First, there is no validated mathematical model to calculate such uneven distributions and signal intensities. Most current analysis methods artificially binarize data first then treat all signal intensities greater then a certain value as an equal event. Clearly, this approach is not accurate. Second, the individual cellular differences make the quantification even more unreliable. Third, the relationship between the reporter intensity and numbers of receptors needs further study. The narrow bands of both excitation sources and emission detectors make it possible to simultaneously detect multiple disease components with optical imaging [30]. The lack of 3D imaging capability in this study and limited signal penetration depth makes it difficult to conduct detailed analysis of disease tissue *in vivo* and to detect disease in deep organs. The future of optical imaging in translational research depends on the development of additional dyes with long penetration depth and 3D reconstruction [31, 32].

The combination of cancer cells with reporters also provides a tool to study tumor biology. Our images vividly show the discordance of signal intensity between luciferase-positive tumor cells and all other imaging agents (Fig. **9**). However, genetically-engineered cell lines, such as cells expressing luciferase or fluorescent proteins, are different from their parental cell lines [33]. These cells continue to change during disease progression and may not represent the true conditions in human disease [28, 34]. Reporters that depend on enzymes for activation have more complicated biochemical requirements, and each parameter has a significant influence on the imaging results. Therefore, enzyme-activated agents provide less accurate information in determination of tumor size than anatomic imaging modalities, such as X-ray, CT, and magnetic resonance imaging [17, 35-37].

Molecular imaging results should always be combined with anatomic images to determine disease location and scope [30]. The greater specificity of the imaging agents will lower the intensity of background signal as low as zero. As the result, it is difficult to determine the location of those signals without anatomic imaging.

An important consideration of molecular targeted approaches is that the imaging agent should never have the potential effect of stimulating the disease. It has been suggested, for example, that reporter labeled EGF can activate the EGFr signaling pathway in breast cancer xenografts [38]. A better imaging agent without biological activity should be developed for human clinical trials to avoid consequences similar to erythropoiesis-stimulating agents [39].

## CONCLUSIONS

We have demonstrated the use of multiple imaging agents and modalities to study tumor characteristics. More importantly, our data support the clinical requirement for multi-agent imaging as a complementary method to interrogate the unique biological information of each individual tumor. We further showed it is possible to separate multiple signals from different tumor components within the same lesion based on different reporters. In addition, the imaging results provide information on disease status at the molecular level. The *in vivo* study illustrated two important aspects of tumor biology and imaging: (1) heterogeneity of the tumor mass and interaction among multiple disease components, and (2) the importance of combining the appropriate imaging modalities to accurately define the relationships of tumor mass, tumor vasculature, and imaging/therapeutic agents. While preclinical experimental conditions are far better controlled than those in human clinical situations, we nevertheless observed significant differences in the signal intensity of each imaging agent and in distribution among animals between time points and from one cell line to another used as xenograft. Long-term longitudinal studies may be required to fully understand the intricacies of any tumor model.

## Figures and Tables

**Fig. (6):**
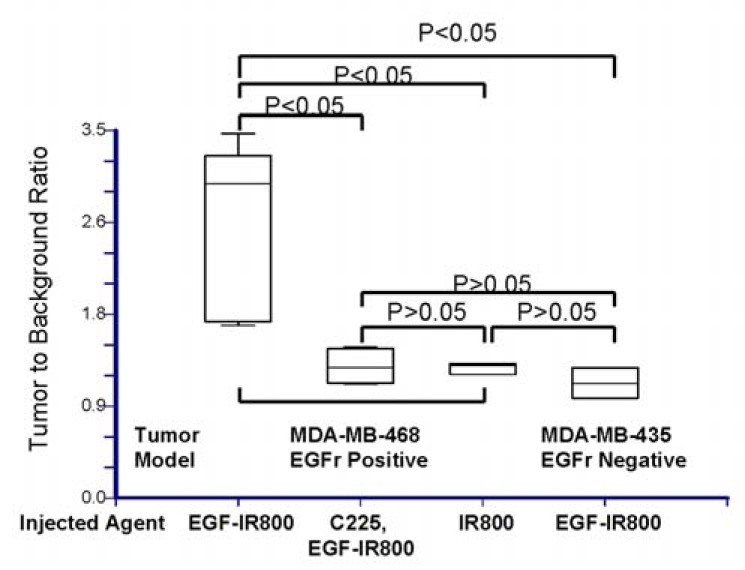
Statistical comparison of TBR from EGF imaging results. The TBR of receptor-positive tumor was significantly higher than the C225 Ab-blocked tumor or the tumor injected with dye alone, as well as the receptor-negative tumor (*P* < 0.05). There were no significant differences between the TRBs in the latter three samples (*P* > 0.05).

## ABBREVIATIONS

Ab: = Antibody
CT: = Computed tomography
DLIA-IL11Rα: = Dual-labeled optical/nuclear IL-11 receptor α agent
EGF: = Epidermal growth factor
EGFr: = Epidermal growth factor receptor
Em: = Emission
Ex: = Excitation
FDA: = United States Food and Drug Administration
FDG or ^18^F-FDG: = Fluorodeoxyglucose
HPLC: = High-performance liquid chromatography
IHC: = Immunohistochemistry
IL-11: = Interleukin 11
MMP: = Matrix metalloproteinase
NIR: = Near-infrared
PET: = Positron emission tomography
SPECT: = Single photon emission computed tomography
TBR: = Tumor-to-background ratio
